# Is high intensity laser therapy more effective than other physical therapy modalities for treating knee osteoarthritis? A systematic review and network meta-analysis

**DOI:** 10.3389/fmed.2022.956188

**Published:** 2022-09-15

**Authors:** Menglai Wu, Lijiang Luan, Adrian Pranata, Jeremy Witchalls, Roger Adams, Jaquelin Bousie, Jia Han

**Affiliations:** ^1^School of Sports and Health, Shanghai University of International Business and Economics, Shanghai, China; ^2^School of Exercise and Health, Shanghai University of Sport, Shanghai, China; ^3^Department of Nursing and Allied Health, School of Health Sciences, Swinburne University of Technology, Melbourne, VIC, Australia; ^4^Research Institute for Sport and Exercise, University of Canberra, Canberra, ACT, Australia; ^5^School of Physiotherapy, University of Sydney, Sydney, NSW, Australia; ^6^Faculty of Health, University of Canberra, Canberra, ACT, Australia; ^7^College of Rehabilitation Sciences, Shanghai University of Medicine and Health Sciences, Shanghai, China

**Keywords:** high intensity laser therapy, physical therapy, knee osteoarthritis, systematic review, network meta-analysis

## Abstract

**Background:**

The use of physical therapy modalities, especially high intensity laser therapy (HILT), for individuals with knee osteoarthritis (KOA) is still controversial.

**Objective:**

To compare the effects of HILT to other physical therapy modalities on symptoms and function in individuals with KOA.

**Methods:**

Six databases (PubMed, Embase, Cochrane Library, Web of Science, EBSCO, and PEDro) were searched in March 2022. Included studies were randomized controlled trials involving HILT conducted on individuals with KOA. The end-trial weighted mean difference (WMD) and standard deviations (SD) with 95% confidence intervals (CI) were analyzed.

**Results:**

Ten studies with 580 participants were obtained, of which nine were included in the final network meta-analysis. In terms of relieving pain, HILT demonstrated the highest probability of being among the most effective treatments, with surface under the cumulative ranking (SUCRA) = 100%, and compared to a control (placebo laser or exercise or a combination of both) on the visual analog scale (VAS) for pain it demonstrated significant benefits (WMD 1.66, 95% CI 1.48–1.84). For improving self-reported function, as measured by the Western Ontario and McMaster Universities Osteoarthritis Index (WOMAC) total scores, the HILT SUCRA value led with 98.9%. When individuals with KOA were treated by HILT, the improvement in stiffness was statistically significant (WMD 0.78, 95% CI 0.52–1.04) but the amount of improvement was smaller than the minimal clinically important difference (MCID).

**Conclusion:**

The current evidence suggests that HILT may be more effective than other physical therapy modalities for improving pain and function in individuals with KOA. For improving stiffness, however, it may not be clinically effective.

**Systematic review registration:**

[https://www.researchregistry.com], identifier [1148].

## Introduction

Knee osteoarthritis (KOA) is a highly prevalent musculoskeletal disorder, occurring mostly in older adults (1). It is characterized by hypertrophy of bone at the joint margins, erosion of the articular cartilage and a wide range of biochemical and morphologic changes in the synovial membrane and joint capsule (1). Clinical manifestations of KOA include pain, joint stiffness, muscle weakness, and limited range of motion (2). Individuals with KOA often encounter difficulties in activities of daily living, such as walking and climbing, and report poor quality of life (3).

The treatment options for KOA include pharmacological, surgical and physical measures (2), while current clinical guidelines primarily recommend non-pharmacological and non-surgical conservative strategies (4, 5). Multifactorial physical therapy modalities have been widely utilized in clinical practice and are believed to be effective for improving symptoms, sport performance, and self-reported function in individuals with KOA (6, 7). These modalities usually involve hot pack treatment, electric stimulation, ultrasound, and combinations of these (4), and studies have found these physical therapy modalities to be beneficial for relieving pain and enhancing activities of daily living (5, 6).

In recent years, laser therapy has been introduced as a physical therapy modality for treating musculoskeletal conditions and has gained popularity since no evident side effects have been reported after intervention (8). Of all the laser therapies, high intensity laser therapy (HILT) is a relatively new type of electrotherapy modality (9). It is a powerful and painless physical modality that has demonstrated significant benefits in antalgic, anti-edema, and biostimulating effects (10). Research has suggested that during HILT, radiation from high intensity laser produces photo-chemical, photothermal, and photomechanical actions (11, 12), especially from neodymium-doped yttrium aluminum garnet (Nd: YAG) laser, which has been found to be powerful in penetrating into deep tissues.

Some reviews have assessed the effects of HILT on symptoms or physical performance in individuals with KOA (9, 13), and two systematic reviews have clearly reported benefits of HILT for pain and function in individuals with KOA using some self-reported scales (9, 14); however, the clinical effectiveness of these outcome measures is still unclear. In addition, little is known about the comparative intervention effects of HILT in relation to other physical therapy modalities. Although previous studies have shown that certain physical therapy modalities, including HILT, seem to be effective for reducing pain and providing physical and functional improvements in individuals with KOA (6, 9); there is yet no consensus about which of currently used physical therapy interventions may have the most benefit for improving symptoms and function in KOA. An answer would be helpful for physiotherapists wanting to select the optimal measurement and intervention in order to provide the most efficient and effective management for KOA.

Accordingly, the aim of current systematic review was to conduct a network meta-analysis (NMA) to compare the effects of HILT and other physical therapy modalities on symptoms and function in individuals with KOA. NMA has been introduced as a generalization of pairwise meta-analysis (15), where indirect effect estimates are calculated with the effect estimates from two comparisons having a common comparator (16); when there is a same object of comparison among the studies, the connections may be built with each other (15). It has been utilized to derive summary comparison measures from a variety of evidence, in order to clarify the effectiveness of one treatment compared to another (17). Additionally, this review aimed to use meta-analysis to further assess the effects of HILT on symptoms and function, and their associated minimal clinically important difference (MCID) values, in persons with KOA.

## Materials and methods

The current study was registered in the Research Registry^1^ (registration no. Reviewregistry1148), in accordance with the Preferred Reporting Items for Systematic Reviews Incorporating Network Meta-Analyses (PRISMA Extension Statement) guidelines and Systematic Reviews and Meta-Analyses (PRISMA 2020 statement) guidelines (18, 19).

### Literature search and selection of studies

A literature search was conducted in March 2022 in PubMed, Embase, Cochrane Library, Web of Science, EBSCO, and PEDro. No restriction was applied on language or the publication year. The search strategy used a mix of MeSH (or Publication Type) and free text terms from the following three key areas: knee osteoarthritis, high intensity laser therapy, and randomized controlled trial.

The entries were set in two steps: (1) In the PubMed database, the key word as “MeSH” was first identified according to the subject, and all relevant items were collected in the “Entry Terms” of “All MeSH Categories” by searching the “MeSH column”; (2) In the Embase database, the key word was again retrieved in the “Emtree column”; if there are new items in the “use preferred term” of “find term,” they would be supplemented. The PubMed search strategy is as follow: (osteoarthritis, knee [MeSH] OR osteoarthritis knee [Title/Abstract] OR knee osteoarthritides [Title/Abstract] OR knee osteoarthritis [Title/Abstract] OR osteoarthritides, knee [Title/Abstract] OR osteoarthritis of knee [Title/Abstract] OR knee, osteoarthritis of [Title/Abstract] OR knees, osteoarthritis of [Title/Abstract] OR osteoarthritis of knees [Title/Abstract]) AND (high intensity laser [MeSH] OR high intensity laser therapy [Title/Abstract] OR high intensity laser therapies [Title/Abstract] OR therapies, high intensity laser [Title/Abstract] OR therapy, high intensity laser [Title/Abstract]) AND (randomized controlled trial [Publication Type] OR randomized [Title/Abstract] OR double-blind [Title/Abstract] OR placebo [Title/Abstract] OR controlled clinical trial [Publication Type] OR controlled [Title/Abstract] OR random [Title/Abstract] OR trial [Title/Abstract]).

Two assessors (M.W. and L.L.) independently assessed all searched studies, screening all titles and abstracts. Full articles of the potential studies were evaluated to obtain eligible trials, and the references in the obtained studies were examined to make sure that all relevant studies were included.

### Inclusion and exclusion criteria

Studies were included by following the PICOS criteria: (1) Participants: individuals with knee osteoarthritis; (2) Interventions: high intensity laser therapy; (3) Comparators: no restriction; (4) Outcomes: no restriction was applied to outcome measures, however, the present network meta-analysis focused on the symptoms and function; (5) Study design: randomized controlled trials.

The exclusion criteria were: (1) Trials performed *in vitro*, with animals, cadavers, simulators, or prosthesis, and those conducted after total knee replacement; (2) Studies that were case reports, or descriptive studies, or not published as peer-reviewed journal articles.

### Literature quality evaluation and bias risk assessment

The literature quality evaluation and risk of bias assessment were conducted independently by two assessors using the Physiotherapy Evidence Database (PEDro) scale and the Cochrane Risk of Bias tool, respectively. Research has shown that both tools are reliable for assessing the quality of studies and evaluating risk of bias (20, 21). The scores for each paper were achieved by the two assessors reaching an agreement through discussion, and any disagreements were resolved by a third reviewer.

Output from the PEDro scale contains 11 items with a total score out of 10 (item 1 is not scored). Each item is scored “no” (0 point) or “yes” (1 point); a score above 6 indicates high quality study, and scores of less than 6 reflect greater potential for biases to affect the results of the trial (22, 23).

Output from the Cochrane Risk of Bias tool is shown as illustrations covering random sequence generation and allocation concealment (selection bias), blinding of participants and personnel (performance bias), blinding of outcome assessment (detection bias), incomplete outcome data (attrition bias), selective reporting (reporting bias), and “other bias.” Each entry is then judged to be at an unclear, low, or high risk of bias (24, 25).

### Data extraction and outcome measures

All data were extracted by two independent reviewers and included; KOA inclusion criteria, participant characteristics (age, sex, height, and weight), HILT or control treatment protocols, the type of laser device, and outcome measures appropriate for analysis. Furthermore, because the purpose of the current study was to compare the effects of HILT and other physical therapy modalities, the HILT intervention was assigned the experimental group, and the control group was any physical therapy modality that was not HILT. Disagreements were resolved by discussion with a third reviewer.

With regard to outcome measures, those of high reliability and practicability, frequently adopted in studies of HILT for KOA were considered. In addition, outcome measures that are representative for the evaluation of knee function in persons with KOA were included. Moreover, they should employ measures suitable for network meta-analysis, presenting baseline and post-intervention data, and able to be compared with their MCID.

The following were documented; the visual analog scale (VAS) (score: 0–10) that evaluates intensity of pain (26), with the MCID for pain in knee osteoarthritis estimated to be 0.9 units (27); and scores from the Western Ontario and McMaster Universities Osteoarthritis Index (WOMAC), that consists of the three sub-scales of pain (0–20), stiffness (0–8), and function (0–68) (a lower score indicates less dysfunction) (28), covering the basic symptoms and characteristics of osteoarthritis, with MCID in KOA being 1.42, 1.30, and 7.65 points respectively (29). MCID values were adjusted for ease of comparison, as the original text adopted the 0–100 scale.

### Data synthesis and statistical analysis

In this review, the outcome measurements (VAS pain points, WOMAC sub-scales, and total scores) were calculated as the mean change score (continuous data) between before and after treatment, with the weighted mean differences (WMD) and standard deviations (SD) [95% confidence intervals (CI)].

When comparing the effects of different physical therapy modalities, STATA was used to perform the network meta-analysis for conducting indirect comparisons. The network of HILT and other physical therapy modalities was presented using a network plot, and the surface under the cumulative ranking (SUCRA) probability was utilized to rank the effectiveness of different physical therapy modalities.

In addition, the meta-analyses were performed using RevMan (Version 5.3) to evaluate the effect of HILT compared to a control such as placebo. The randomized effects model with the inverse variance method was used, and I^2^ tests were applied to evaluate the statistical heterogeneity (values exceeding 50% implied a moderate to high heterogeneity), and a *p*-value less than 0.05 taken to indicate a statistically significant difference.

## Results

### Literature search and screening

**FIGURE 1:**
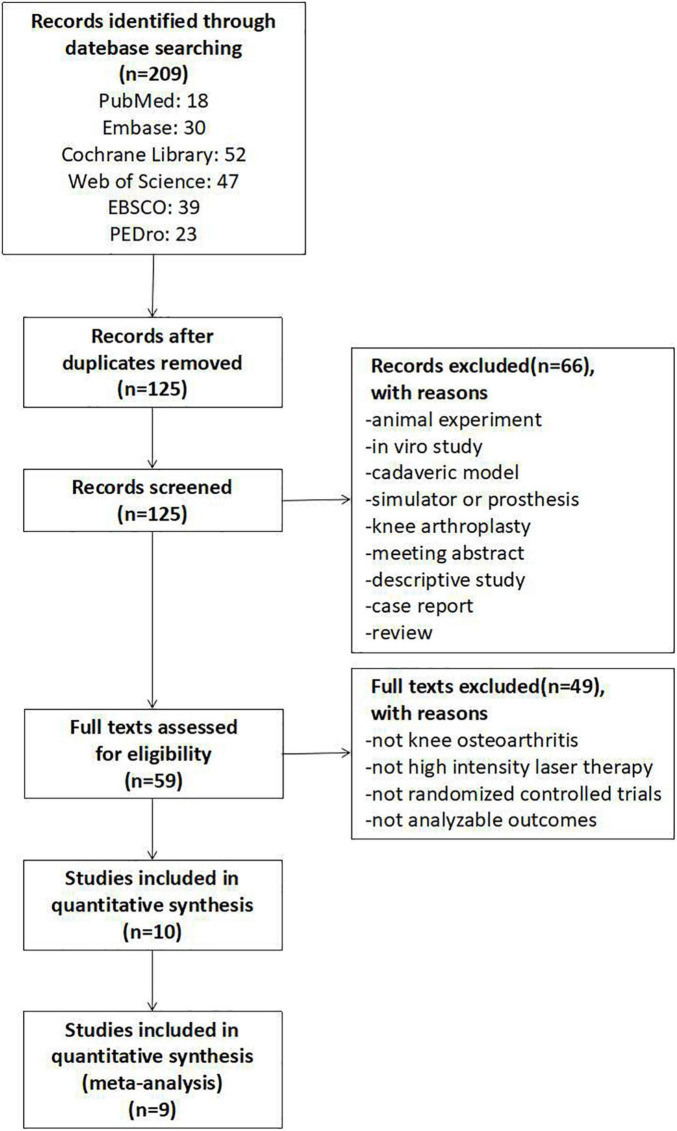
Flowchart of study selection.

A total of 209 relevant studies from the six electronic databases were obtained, and 10 RCTs involving 580 participants were selected (30–39), of which 9 were included in the final network meta-analysis (31–39). The one study not included had no post-intervention outcome measures (30). The detailed selection process is shown in Figure 1.

### Study quality and risk of bias

For all included studies, the average score on the PEDro scale, which ranges from 5 to 8, was 6.3, and the quality evaluation details are presented in Table 1. The most frequent shortcomings of the obtained trials were: unclear description of concealed allocation, ambiguous information about the outcome measures, and inadequate blinding methods. Overall, the quality of the included studies was moderate.

**TABLE 1:** Physiotherapy evidence database (PEDro) scores of included studies.

Study	Eligibility criteria	Random allocation	Concealed allocation	Groups similar at baseline	Participant blinding	Therapist blinding	Assessor blinding	<15% dropouts	Intention to treat analysis	Between group difference reported	Point estimate and variability reported	Total (0–10)
Akaltun et al. (30)	Yes	Yes	Yes	Yes	Yes	Yes	No	Yes	Yes	No	No	7
Alayat et al. (31)	Yes	Yes	Yes	Yes	No	No	No	Yes	No	Yes	Yes	6
Angelova and Ilieva (32)	Yes	Yes	No	Yes	Yes	No	No	Yes	Yes	Yes	Yes	7
Delkhosh et al. (33)	Yes	Yes	No	Yes	Yes	No	No	No	No	Yes	Yes	5
Gworys et al. (34)	Yes	Yes	No	Yes	No	No	No	Yes	No	Yes	Yes	5
Kheshie et al. (35)	Yes	Yes	Yes	Yes	Yes	No	No	Yes	No	Yes	Yes	7
Kim et al. (36)	Yes	Yes	No	Yes	No	No	No	Yes	No	Yes	Yes	5
Kim et al. (37)	Yes	Yes	No	Yes	Yes	Yes	No	No	No	Yes	No	5
Mostafa et al. (38)	Yes	Yes	Yes	Yes	No	Yes	Yes	Yes	Yes	No	Yes	8
Nazari et al. (39)	Yes	Yes	Yes	Yes	Yes	Yes	Yes	No	No	Yes	Yes	8

**FIGURE 2:**
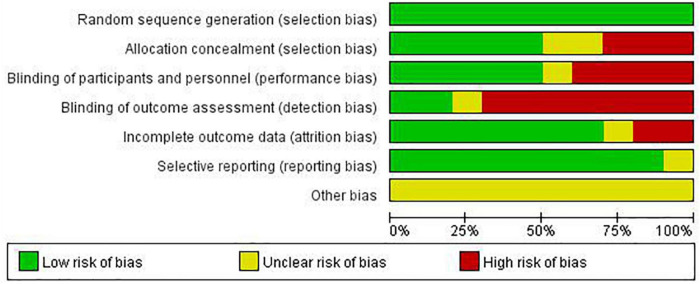
Risk of bias graph.

The summary risk of bias assessment with the Cochrane Collaboration criteria is reported in Figure 2. The included studies all described a low risk of bias within the process of random sequence generation. However, the allocation concealment and outcome data were unclear in several trials (32–34, 36, 37). In addition, some studies were classified as having high risk of bias in performance and detection because the researchers participated in the interventions and/or assessments (30–37), or the participants were not blinded to the treatment (31, 34, 36, 38).

### Characteristics of the eligible studies

The included studies were published from 2009 to 2022 (30–39). All participants had a diagnosis of KOA, and their characteristics and measures of intervention are summarized in Table 2.

**TABLE 2:** Characteristics of included studies.

Studies	Inclusion criteria of KOA	High-intensity laser therapy			Control		Analyzable outcomes
			
		Participants	Treatments	HIL device	Participants	Treatments	
Akaltun et al. (30)	(1) Grade II-III KOA cases according to K-L classification; (2) being aged between 45 and 75; (3) having knee pain for at least 6 months; and (4) the VAS score being at least 3 or more.	*n* = 20 age (year): 57.85 ± 8.07 gender (M/F): 6/14 BMI (Kg/m^2^): 29.94 ± 5.49	The analgesic mode was applied on the first 3 days. A total of 300 J was applied as 12 J/m^2^ 25 cm^2^ at a frequency of 25 Hz in these sessions. The biostimulation mode was implemented as of the fourth session. A total of 3,000 J was applied as 120 J/cm^2^ in this mode. The application was made continuously with circular motions in both modes. (A total of 10 sessions were implemented)	Nd:YAG Laser with 1,064 nm wavelength (BTL-6000 HIL 12 W)	*n* = 20 age (year): 58.62 ± 11.28 gender (M/F): 6/14 BMI (Kg/m^2^): 31.95 ± 4.87	Placebo laser: The same device was applied in the form of a sham laser that did not radiate laser beam. Exercise: The home exercise program included ROM exercises, stretching, strengthening, and flexibility exercises.	Null
Alayat et al. (31)	(1) Degenerative osteoarthritic knee of grade III or less based on K-L classification; (2) persistent pain ≥ 4 on the VAS for more than 3 months in one or both knees; (3) BMI ≤ 30 kg/m^2^; and (4) self-reported disability due to knee pain with a score of at least 25 on the WOMAC.	*n* = 23 age (year): 55 ± 4.41 gender (M/F): all male height (cm): 171.9 ± 3.46 weight (kg): 83.21 ± 5.82 BMI (Kg/m^2^): 28.15 ± 1.72	Each anterior or posterior knee surface was scanned in two sub-phases (initial and final) with three fluency levels. Initially, fast scanning was performed with gradually increasing fluency in three levels: level I: with energy density of 1,430 mJ/cm^2^, frequency of 30 Hz, in 30.4 s; level II: with energy density of 1,530 mJ/cm^2^, frequency of 25 Hz in 34 s; and level III: with energy density of 1,780 mJ/cm^2^, frequency of 20 Hz, and time of application 36.6 s. In each level, 250 J was delivered with a total of 750 J for anterior knee surface, and the same sequence was performed to posterior knee surface. The final phase was similar to the initial phase except that the scanning was slow. The average area was 200 cm^2^ and the average energy density was 15 J/cm^2^, with a total energy of 3,000 J delivered in each session. (2 times per week for 6 weeks)	Pulsed Nd:YAG laser (HIRO 3.0, ASA, Arcugnano, Vicenza, Italy)	*n* = 22 age (year): 52.86 ± 5.03 gender (M/F): all male height (cm): 170.95 ± 4.64 weight (kg): 83.68 ± 4.50 BMI (Kg/m^2^): 28.63 ± 1.00	Placebo laser: A sham laser was administered in the same way as the active laser. Exercise: The program included ROM exercise, flexibility, stretching, and strengthening training.	VAS WOMAC (pain, stiffness, function)
					*n* = 22 age (year): 53.64 ± 3.54 gender (M/F): all male height (cm): 171.64 ± 3.82 weight (kg): 85.18 ± 3.42 BMI (Kg/m^2^): 28.91 ± 0.49	Medication: patients received glucosamine sulfate potassium chloride and chondroitin sulfate sodium 3 times daily.	
Angelova and Ilieva (32)	(1) Duration of the symptoms for over 4 years and X-ray stages II and III by K-L; (2) without local application of corticosteroids or hyaluronic acid during the last 6 months; (3) without physiotherapy during the last 6 months; and (4) being treated with physiotherapy or drugs more than 6 months before.	*n* = 35 age (year): 65.11 ± 1.4 gender (M/F): 11/24	The first three procedures are with analgesic effect with dose 12 J/cm^2^ = 300 J for treated area of 25 cm^2^. Laser therapy is applied on the medial and lateral sides of the knee, distant application, for 2 min, 25 Hz frequency. The next 4 sessions use biostimulating parameters, applied with dose 120 J/cm^2^ = 3,000 J treated area 25 cm^2^, applied on the medial side of the knee, 10 min. (A total of seven sessions were implemented)	Semiconductive neodymium laser IV with wave length 1,064 nm and maximal power 12 W (BTL)	*n* = 37 age (year): 64.71 ± 1.98 gender (M/F): 12/25	Placebo laser: The patients are treated with imitation of laser treatment by directing the laser device without turning on the light beam (sham laser).	VAS
Delkhosh et al. (33)	The condition of patients with KOA was based on a diagnosis of specialists or doctors from Orthopedics for Physiotherapy Centers of the University of Medical Sciences.	*n* = 15 age (year): 43.5 ± 8.4 gender (M/F): all female height (cm): 154.1 ± 5.5 weight (kg): 58.5 ± 4.8	The Ga-A1-As laser with maximal power of 5 W and wave length of 1,064 nm were used on 4 points (anterior and inside of the knee joint). Each session was divided into three phases, and each phase was 4 min. The total energy was approximately 1,400 J per session.	Ga-A1-As laser	*n* = 15 age (year): 48.3 ± 4.0 gender (M/F): all female height (cm): 150.5 ± 4.4 weight (kg): 70.5 ± 1.0	Placebo laser: The patients received inactive laser (the device was turned off).	VAS WOMAC (total)
					*n* = 15 age (year): 44.5 ± 8.7 gender (M/F): all female height (cm): 150.3 ± 4.5 weight (kg): 58.3 ± 1.0	Low level laser therapy: The laser Ga-Al-As with power of 300 mW and wave length of 830 nm were used on 4 points (anterior and inside of the knee joint). Dosage: average energy density was 50 J/cm^2^ per point.	
Gworys et al. (34)	The study involved patients with pain of more than 6 weeks’ duration and a diagnosis of KOA according to the criteria established by the American College of Rheumatology. Enrollment criteria also included 2nd degree joint injury according to Seyfried on the basis of clinical examination, no intraarticular corticosteroids, hyaluronic acid or other drugs within the 3 months preceding the study, no physical therapy during the 3 months, and no contraindications for physical therapy.	*n* = 30 age (year): 65.4 ± 9.6	The patients received two-wave laser irradiation (power 1,100 mW, frequency 2,000 Hz, dose 12.4 J/point, energy density 6.21 J/cm^2^). The knee joint was irradiated in 12 points: three points each at the level of the medial and lateral aspect of the knee joint gap, two points each at the level of the patellofemoral joint on the superior and inferior aspect of the joint, and two points in the popliteal fossa. Laser therapy sessions were performed once a day, 5 days a week over 2 weeks. Each patient attended 10 sessions.	Multiwave Locked System (MLS) Therapy (synchronized generation of continuous (wave length 808 nm) and pulsed (wave length 905 nm) laser light)	*n* = 31 age (year): 67.7 ± 11.3	Placebo laser: Laser therapy procedures were simulated without actual irradiation.	VAS
					*n* = 34 age (year): 57.6 ± 11.8	Low level laser therapy: The patients received one-wave laser irradiation (wave length 810 nm, dose 8 J/point, surface density of energy 12.7 J/cm^2^, power 400 mW, and surface density of power 634.9 mW/cm^2^) in the continuous mode.	
					*n* = 30 age (year): 65.9 ± 9	Mild dose laser therapy: The patients received two-wave laser irradiation (power 1,100 mW, frequency 2,000 Hz, dose 6.6 J/point, energy density 3.28 J/cm^2^).	
Kheshie et al. (35)	(1) Had painful KOA for at least 6 months with degenerative osteoarthritic knee of grade II-III or less based on radiographic diagnosis in the K-L grading of osteoarthritis; (2) had no limitation of range of motion except for minimum tightness in the knee joint; (3) did not engage in any high-joint-loading exercises such as hiking or tennis playing and had not undergone any specific treatments 3 months before entering the study; (4) had a minimum score of 25 on the WOMAC total score, and (5) had a knee pain ≥ 4 on the VAS in the previous 3 months.	*n* = 20 age (year): 52.1 ± 6.47 gender (M/F): all male height (cm): 172 ± 5.49 weight (kg): 88.55 ± 7.51 BMI (Kg/m^2^): 29.94 ± 3.36	The scanning was performed transversely and longitudinally in the anterior, medial, and lateral aspects of the knee joint with emphasis on the application on the joint line between the tibial and femoral epicondyles. The total energy delivered to the patient during one session was 1,250 J through three phases of treatment. The initial phase was performed with fast manual scanning with a total of 500 J. In the initial phase, the laser fluency was set to two successive sub-phases of 710 and 810 mJ/cm^2^ for a total of 500 J. In the intermediate phase, the handpiece was applied on the joint line just proximal to the medial and lateral tibial condyles with 25 J, a fluency of 610 mJ/cm^2^, and a time of 14 s for each point and a total of 250 J in this phase. The final phase was the same as the initial phase except that scanning was slow manual scanning. The application time for all three phases was approximately 15 min with the total energy delivered to the patient during one session of 1,250 J. (two sessions per week for 6 weeks)	Pulsed Nd:YAG laser (HIRO 3.0, ASA, Arcugnano, Vicenza, Italy)	*n* = 15 age (year): 55.6 ± 11.02 gender (M/F): all male height (cm): 175 ± 6.30 weight (kg): 87.00 ± 7.75 BMI (Kg/m^2^): 28.51 ± 3.35	Placebo laser: Patients attended the physical therapy clinic two times a week for 6 weeks and received sham laser. Exercise: Patients received an exercise program which consisted of active ROM exercises, muscle strengthening, and flexibility exercises.	VAS WOMAC (pain, stiffness, function)
					*n* = 18 age (year): 56.56 ± 7.86 gender (M/F): all male height (cm): 173 ± 4.92 weight (kg): 85.16 ± 14.03 BMI (Kg/m^2^): 28.62 ± 5.20	Low level laser therapy: Patients received gallium-arsenide diode (GaAs) laser (BTL-5000 laser) infrared probes with a wavelength of 830 nm, output power of 800 mW, average energy density of 50 J/cm^2^, frequency of 1 KHz, and duty cycle of 80%. The cluster laser was in direct contact and perpendicular to the affected knee with a time of application of 32 min and 33 s per session and a total energy of 1,250 J. (two sessions per week for 6 weeks)	
Kim et al. (36)	The subjects’ attending doctors had diagnosed them with KOA based on clinical findings and images taken using X-ray equipment.	*n* = 10 age (year): 65.3 ± 4.2 height (cm): 159.3 ± 7.4 weight (kg): 62.0 ± 11.0	A high intensity laser was applied in the tibia and femoral epicondyle for 5 min. A separation distance of around 1 cm between the handpiece and the skin was also maintained throughout the treatment. The intensity of the HIL was level 2, the frequency was 11 Hz, and the total amount of delivered energy was 1,500 mJ/cm^2^. (3 times per week for 4 weeks)	HEALTRON (United Technology Inc., Israel)	*n* = 10 age (year): 65.5 ± 4.0 Height (cm): 159.9 ± 8.2 weight (kg): 61.6 ± 10.4	Thermotherapy plus interferential current: The program consisted of hot pack treatment for 20 min, interferential current therapy for 15 min, and deep heat diathermy using ultrasonic waves for 5 min.	VAS WOMAC (total)
Kim et al. (37)	The subjects had grade II osteoarthritis of Kellgren classification and knee pain.	*n* = 14 age (year): 60.6 ± 2.98	Patients underwent treatment 30 times. Three sub-phases of high intensity laser, 500 J of the energies were evenly transferred through each phase 60 s. (A) For the anterior condyle of the femur, the internal and external hematoma of the knee flexed at 90° on supine position. (B) For the posterior side of the patella, the lateral and medial windows at the 30° knee flexion state. (C) For the posterior condyle of the femur, the internal and external hematoma of the knee over the popliteal fossa at the maximum knee extension state. (5 times per week for 6 weeks)	Pulsed Nd:YAG laser (HIRO 3.0, ASA, Arcugnano, Vicenza, Italy)	*n* = 14 age (year): 61.6 ± 2.98	Placebo laser: The same device was applied in the form of a sham laser that did not radiate laser beam.	VAS WOMAC (pain, stiffness, function)
Mostafa et al. (38)	Patients were diagnosed with chronic KOA according to the American College of Rheumatology criteria, and they had stage II KOA measured by X-ray, according to Kellgren and Lawrence.	*n* = 20 age (year): 46.62 ± 8.68 height (cm): 163.2 ± 7.3 weight (kg): 77.17 ± 6.38 BMI (Kg/m^2^): 29.26 ± 2.48	Participants received high-intensity pulsed Nd:YAG laser therapy at a frequency of 30 Hz and total delivered energy of 1,500 mJ/cm^2^ in each session, three sessions/week for 4 weeks. To expose the joint surfaces to the laser beam, the HILT handpiece was positioned in contact with and perpendicular to the medial side of the knee while the patient lay supine with the knee flexed at 30° (optical windows). The HILT handpiece was then moved transversely and longitudinally in the anterior, medial, and lateral aspects of the knee joint, emphasizing the joint line between the tibial and femoral epicondyles.	Pulsed Nd:YAG laser (HIRO 3.0, ASA, Arcugnano, Vicenza, Italy)	*n* = 20 age (year): 40.12 ± 9.45 height (cm): 162.2 ± 7.1 weight (kg): 76.02 ± 10.23 BMI (Kg/m^2^): 28.82 ± 5.23	Shock wave therapy: Patients received 1,000 extracorporeal shock wave pulses (Evotron RFL0300 Focal Shockwave; manufactured by Swiss Tech Medical AG, Switzerland) with an intensity of 0.05 mJ/mm^2^, one session/week for 4 weeks. Shock wave therapy is applied to the tender point of the medial tibial plateau area in the affected knee while the patient is lying in a supine position with knees bent at 90°.	VAS WOMAC (total)
Nazari et al. (39)	(1) X-ray stages II and III osteoarthritis according to the criteria proposed by K-L; (2) age between 50 and 75 years; (3) BMI equal to or less than 30; (4) knee pain lasted at least 6 months with intensity at least 3 on VAS scale in activities such as going up- and downstairs, sitting and squatting; (5) no history of acute traumatic injuries; (6) no history of previous surgery or injury in the knee and lower extremities; (7) lack of neuromuscular disease; (8) normal mental state; (9) absence of bone implants; (10) no history of new fractures; (11) lack of cancerous tumors; (12) no history of chronic disease and any condition that affect the study; (13) not participating in sports programs and physical therapy in the recent 3 months; and (14) no history of knee intra-articular injection in the past 6 months.	*n* = 30 age (year): 61.5 ± 3.9 gender (M/F): 13/17 BMI (Kg/m^2^): 27.7 ± 1.4	The treatment was performed in a slow manual scanning in longitudinal and perpendicular direction on the medial and lateral sides of the knee with a 6-cm probe. The probe was placed vertically in contact with the joint line while the patient was in a supine position and the knee flexed at 30° for 8 min, at a frequency of 30 Hz with a peak power of 5 W, a duty cycle of 70%, energy density of 60 J/cm^2^, and total energy of 2,400 J during one session. (3 times per week for 4 weeks)	Pulsed mode of E20780–Nd:YAG laser with wavelength of 1,064 nm (Fysiomed, Belgium)	*n* = 30 age (year): 62.4 ± 3.14 gender (M/F): 14/16 BMI (Kg/m^2^): 27.2 ± 1.6	Electric stimulation plus ultrasound: The participants were treated by a special equipment, including transcutaneous electric nerve stimulation (TENS) and ultrasound (US) for 12 sessions, on alternate days. The former was delivered by two self-adhesive electrodes which were placed on the medial and lateral parts of the knee joint line, which was applied to the patients using a frequency of 100 Hz, pulse width of 50–100 μs, and quadratic biphasic symmetrical pulse shape for 20 min, and the intensity (mA) was set at the individual threshold of a tingling sensation. The latter protocol consisted of continuous ultrasonic waves of 1 MHz frequency and 1 W/cm^2^ intensity applied with a 5 cm diameter applicator. The patients were placed in a supine position, and the ultrasound was applied to the medial and lateral parts (5 min on each side) of the knee in circular movements with the probe at right angles to ensure maximum absorption of the energy.	VAS WOMAC (pain, stiffness, function, total)
					*n* = 30 age (year): 62.24 ± 3.87 gender (M/F): 14/16 BMI (Kg/m^2^): 27.5 ± 1.8	Exercise: The program consisted of nine exercises as follows: walking at the usual speed on a flat surface, hamstring and calf gentle stretches, straight leg raise, quadriceps setting, pillow squeeze, heel raise, one leg balance, step ups, and quadriceps strengthening.	

KOA, knee osteoarthritis; HIL, high-intensity laser; K-L, Kellgren-Lawrence; ROM, range of motion; BMI, body mass index; Nd:YAG, Neodymium:Yttrium Aluminum Garnet; VAS, visual analog scale; WOMAC, Western Ontario and McMaster Universities Osteoarthritis Index.

In terms of high intensity laser therapy, the devices employed and programs adopted were different between trials, however, the main components of each protocol could be identified. With regard to the control group, in this review placebo laser or exercise or a combination of both were classified into one category—placebo laser (plus exercise)—since the placebo laser was almost completely ineffective and exercise was only an auxiliary measure in the included studies when it was used as a control. For other physical therapy modalities, the classification was based on the treatment characteristics as described in the original study.

Specifically, treatments analyzed included eight for placebo laser (plus exercise) (30–35, 37, 39), ten for HILT (30–39), three for low level laser therapy (33–35), one for mild dose laser therapy (34), one for thermotherapy plus interferential current (36), one for shock wave therapy (38), and one for electric stimulation plus ultrasound (39).

### Network meta-analysis of high intensity laser therapy vs. other physical therapy modalities on visual analog scale pain

**FIGURE 3:**
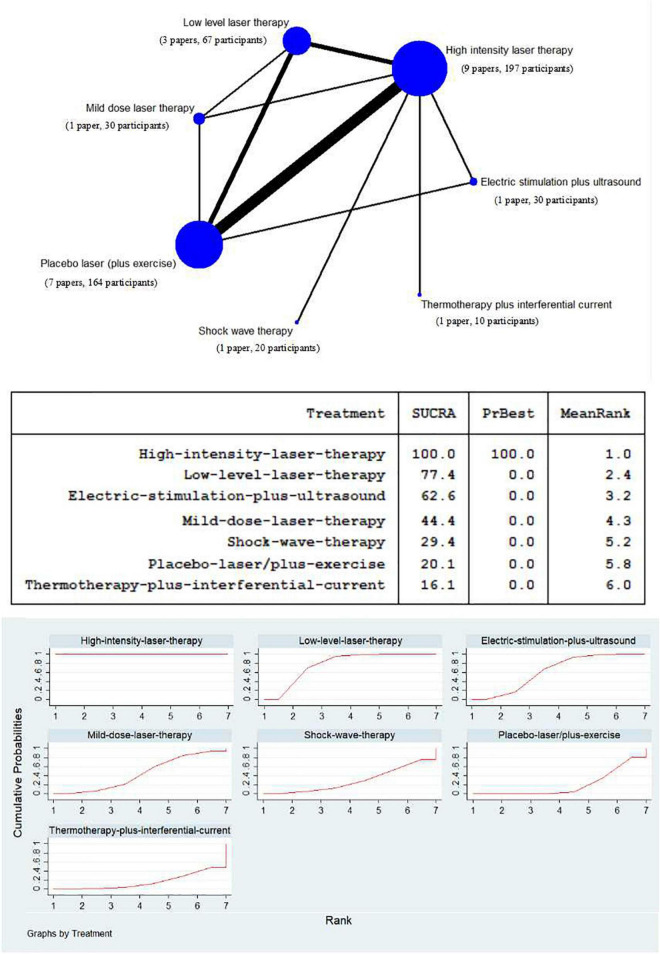
Network meta-analysis plot for the assessment of high intensity laser therapy (HILT) and other physical therapy modalities (nodes are weighted in accordance with the number of trials including the respective treatments. The larger the size of node and the thicker the lines are, the more studies are involved). Treatment relative ranking [the PrBest means the estimated probability that the treatment is the best one. The lower the value of Mean Rank is, the higher the efficacy of the treatment may be. The ranking probability plot for the assessment of improved visual analog scale (VAS) pain at the end of the physical therapy modalities is shown].

To assess the efficacy of HILT and other physical therapy modalities for relieving pain in persons with KOA, a network of treatment was conducted, consisting of seven competing interventions (Figure 3). Except for high intensity laser therapy with 197 participants and placebo laser (plus exercise) with 164 participants, there are relatively more studies using low level laser therapy, with 67 participants; while the experiments of adopting mild dose laser therapy, thermotherapy plus interferential current, shock wave therapy, and electric stimulation plus ultrasound were all with 30 or fewer subjects.

The SUCRA data synthesis (Figure 3) showed that high intensity laser therapy had the highest probability (100.0%) of being among the most effective treatments. In addition, low level laser therapy was superior to mild dose laser therapy, where the former was at 77.4% and the latter at 44.4%. Furthermore, with regard to other physical therapy modalities, apart from placebo laser (plus exercise) (20.1%), electric stimulation plus ultrasound, shock wave therapy, and thermotherapy plus interferential current stood at 62.6, 29.4, and 16.1%, respectively.

### Meta-analysis of high intensity laser therapy vs. low level laser therapy on visual analog scale pain

**FIGURE 4:**
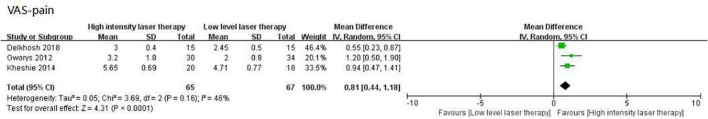
Forest plot of the visual analog scale (VAS)-pain in high intensity laser therapy (HILT) vs. Low level laser therapy.

When compared to low level laser therapy, the effect of HILT measured by VAS pain scores was significantly greater (WMD: 0.81, 95% CI: 0.44–1.18, *I*^2^ = 46%, *p* < 0.0001; Figure 4).

### Meta-analysis of high intensity laser therapy vs. placebo laser (plus exercise) on visual analog scale pain

**FIGURE 5:**
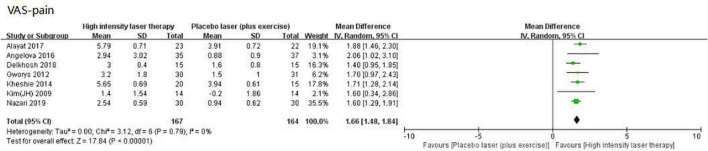
Forest plot of the visual analog scale (VAS)-pain in high intensity laser therapy (HILT) vs. Placebo laser (plus exercise).

High intensity laser therapy was significantly superior to control [placebo laser (plus exercise)] when compared using outcomes assessed by VAS pain scores (WMD: 1.66, 95% CI: 1.48–1.84, *I*^2^ = 0%, *p* < 0.00001; Figure 5), with an effect which exceeded its MCID value of 0.9 (Table 3).

**TABLE 3:** Comparison of intervention effects and MCID.

Outcome	MCID	High intensity laser therapy Vs. Placebo laser (plus exercise)	
		
		*Change scores	^#^Clinical decision
VAS-pain	0.9	1.66 (1.48, 1.84)	Very likely
WOMAC-pain	1.42	2.74 (2.41, 3.08)	Very likely
WOMAC-stiffness	1.30	0.78 (0.52, 1.04)	Very unlikely
WOMAC-function	7.65	8.37 (6.90, 9.85)	Likely
WOMAC-total	10.37	10.87 (8.85, 12.88)	Likely

MCID, minimal clinically important difference; VAS, visual analog scale; WOMAC, Western Ontario and McMaster Universities Osteoarthritis Index. *Values are weighted mean difference (95% confidence interval). ^#^Likely to obtain an effect of the stated size when intervention is used. Very likely, The mean of change scores outweigh MCID, and the lower bound of the 95% CI is more than the MCID. Likely, The mean of change scores outweigh MCID, but the lower bound of the 95% CI is less than the MCID. Very unlikely, The mean of change scores below MCID, and the upper bound of the 95% CI is less than the MCID.

### Network meta-analysis of high intensity laser therapy vs. other physical therapy modalities on Western Ontario and McMaster Universities Osteoarthritis Index

**FIGURE 6:**
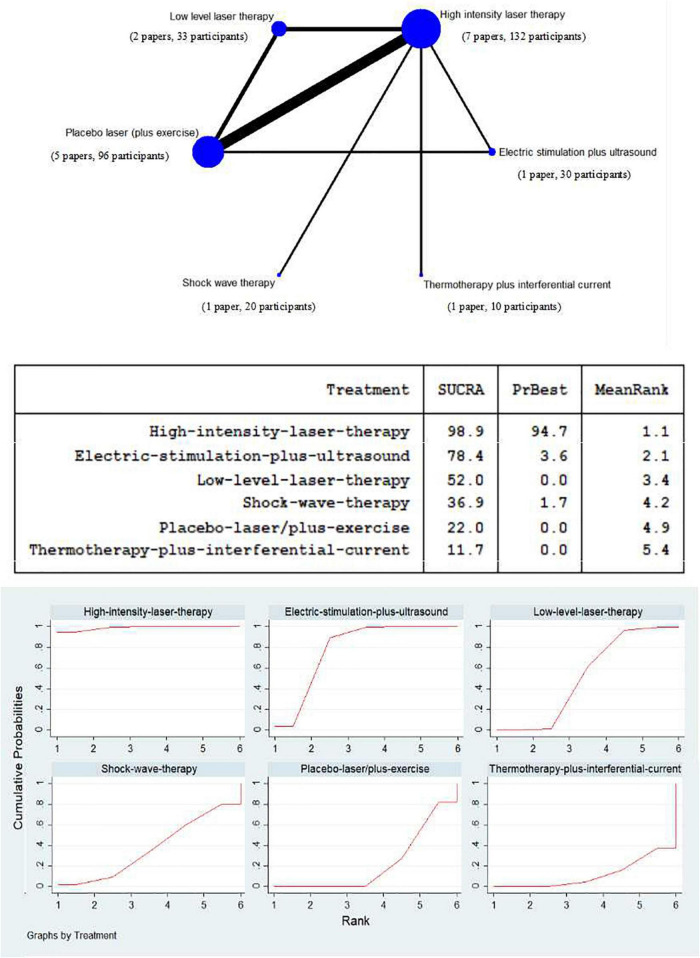
Network meta-analysis plot for the assessment of high intensity laser therapy (HILT) and other physical therapy modalities (nodes are weighted in accordance with the number of trials including the respective treatments. The larger the size of node and the thicker the lines are, the more studies are involved). Treatment relative ranking (the PrBest means the estimated probability that the treatment is the best one. The lower the value of Mean Rank is, the higher the efficacy of the treatment may be. The ranking probability plot for the assessment of improved WOMAC total at the end of the physical therapy modalities is shown).

Due to the limitation of insufficient number of studies with three WOMAC sub-scales (only four studies), the WOMAC total scores of the eligible studies were counted to conduct a network of treatment, comparing the efficacy of HILT and other physical therapy modalities in terms of improving symptoms and function in individuals with KOA, which consisted of six competing interventions as follows: 132 participants of high intensity laser therapy, 96 participants of placebo laser (plus exercise), 33 participants of low level laser therapy, 30 participants of electric stimulation plus ultrasound, 20 participants of shock wave therapy, and 10 participants of thermotherapy plus interferential current (Figure 6).

The SUCRA value of high intensity laser therapy, at 98.9%, was the highest in the compared treatments. The next two were electric stimulation plus ultrasound (SUCRA = 78.4%) and low level laser therapy (SUCRA = 52.0%). The SUCRA values of shock wave therapy, placebo laser (plus exercise), and thermotherapy plus interferential current were at relatively low levels, and scored 36.9, 22.0, and 11.7%, respectively (Figure 6).

### Meta-analysis of high intensity laser therapy vs. low level laser therapy on Western Ontario and McMaster Universities Osteoarthritis Index

**FIGURE 7:**
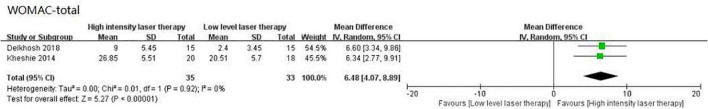
Forest plot of the Western Ontario and McMaster Universities Osteoarthritis Index (WOMAC)-total in high intensity laser therapy (HILT) vs. Low level laser therapy.

Compared to low level laser therapy, when HILT was used there was greater improvement as reflected by the WOMAC total, with a difference which was statistically significant (WMD: 6.48, 95% CI: 4.07–8.89, *I*^2^ = 0%, *p* < 0.00001; Figure 7).

### Meta-analysis of high intensity laser therapy vs. placebo laser (plus exercise) on Western Ontario and McMaster Universities Osteoarthritis Index

**FIGURE 8:**
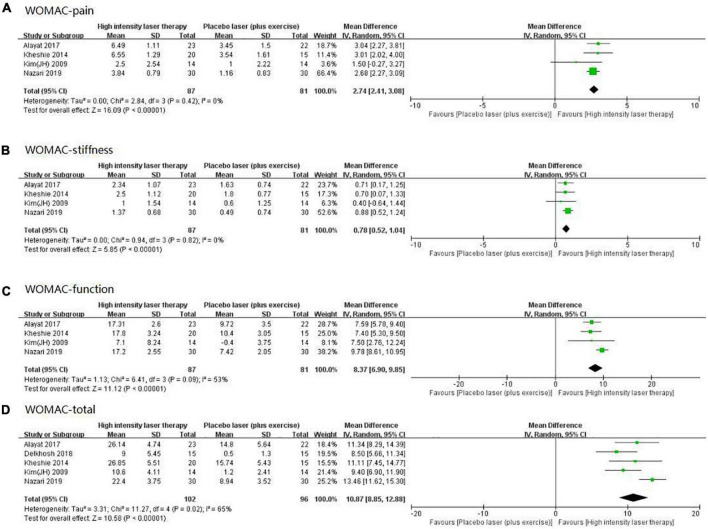
Forest plot of the Western Ontario and McMaster Universities Osteoarthritis Index (WOMAC)-pain (A), WOMAC-stiffness (B), WOMAC-function (C), and WOMAC-total (D) in high intensity laser therapy (HILT) vs. Placebo laser (plus exercise).

High intensity laser therapy demonstrated several benefits as measured by the WOMAC, with findings for its three sub-scales and total scores shown in Figure 8, all of which were statistically significant: (1) pain (WMD: 2.74, 95% CI: 2.41–3.08, *I*^2^ = 0%, *p* < 0.00001; Figure 8A); (2) stiffness (WMD: 0.78, 95% CI: 0.52–1.04, *I*^2^ = 0%, *p* < 0.00001; Figure 8B); (3) Function (WMD: 8.37, 95% CI: 6.90–9.85, *I*^2^ = 53%, *p* < 0.00001; Figure 8C); (4) Total (WMD: 10.87, 95% CI: 8.85–12.88, *I*^2^ = 65%, *p* < 0.00001; Figure 8D). The comparison of these meta-analysis results and their relevant MCID values are presented in Table 3.

## Discussion

The present network meta-analysis showed that the effect of HILT may be superior to other physical therapy modalities in the management of KOA. Although the results of the meta-analysis demonstrated that HILT was more effective than placebo laser (plus exercise), as judged by the WMD for VAS pain as well as for the WOMAC sub-scales and total, for WOMAC stiffness the WMD value did not reach its MCID.

In addition, a previous systematic review (14) was noteworthy, which conducted the meta-analysis, including some studies that are the same as those included in this review. However, there are some significant differences between the paper and this review. First, the previous review included 6 studies; while this study included 10 articles, and 9 of them were involved in meta-analysis. There were certain new information in the additional four studies (30, 33, 36, 38). Also, there was no language restriction in this systematic review; for example, this review included an article (33) that was written in Persian. Most importantly, this review did not only analyze the outcomes of comparing HILT with control, while it actually focused on comparing HILT to other existing physical therapy modalities for treating KOA by a network meta-analysis, which was aimed at drawing a guiding recommendation for therapists to choose physical therapy modality in the management of KOA. This was not available in previous studies.

Admittedly, comparing HILT to control, the data included in this review were not much more than that in the previous review. Only three studies (33, 36, 38) (105 participants) were added in meta-analysis (40 subjects, 45 subjects, and 20 subjects, respectively), which may not cause significant changes on the effects of HILT for individuals with KOA comparing to previous studies. However, the clinical effectiveness of HILT could be found in this study, which was previously unavailable, by comparing the result of meta-analysis with the MCID; moreover, this systematic review and meta-analysis is that the most studies were included at present, and its sample size and level of evidence are the most up-to-date and complete review of this body of literature.

### The effect of high intensity laser therapy on pain

High intensity laser such as Pulsed Nd:YAG has a high peak power laser (3 kW) with a wavelength (up to 1,000 nm), and can deliver sufficient dosage to reach deep target tissue, which has a positive effect on reducing pain in various types of musculoskeletal pain (31). Moreover, HILT that has several photochemistry effects projects a strong optical energy into the tissues where it can generate oscillatory stimulation, which has been demonstrated to improve tissue metabolism and blood circulation (37). This results in a rapid removal of inflammatory substances, improvement of mitochondrial oxidation and production of adenosine triphosphate that help to facilitate more efficient absorption of tissue edema (36, 37).

Additionally, due to its biostimulation, analgesic, and anti-inflammatory effects, HILT is a modality that can be used in many painful conditions (32, 39). The physiological mechanism by which HILT provides pain relief is proposed to occur through the necrotic cells being subjected to extremely high temperature, then exfoliated for a short time (32, 40). This laser application increases local blood circulation in joints, promotes exchange of nutrients within the cartilage, improves tissue regeneration, and consequently seems to be helpful in eliminating inflammation and reducing pain and edema (36, 39, 41).

Compared to other currently available physical therapy modalities as a control, HILT seems to be the most effective treatment for relieving pain in individuals with KOA (SUCRA = 100.0%). The second most effective treatment may be low level laser therapy (LLLT), which uses low intensity laser that has the potential to produce photochemical reactions and improve the metabolism of cells, without causing heat, in order to stimulate or inhibit the cells (42). LLLT has been used with several different diseases, mainly for pain relief (42). Moreover, LLLT is a safe and non-invasive method which has recently attracted the attention of many researchers interested in modalities for treatment of KOA (42).

In previous studies, LLLT has been found to effectively relieve pain and enhance function for individuals with KOA, and these were significant findings (42, 43). LLLT as a therapeutic approach does not release heat, does not damage tissue, and has a relatively mild amount of energy delivered to target tissues (43). The low penetration of low level laser, with power density (below 5 W/cm^2^) and wavelength (540–830 nm), has been widely shown to be secure and stable (42), and most of the technologies used in applications such as scanning, points, and acupuncture are already well-established in musculoskeletal conditions such as pain and arthritis (44). Many studies have also reported a significant effect of LLLT in reducing periarticular inflammation and swelling, as well as for enhancing knee microcirculation, ambulation, and quality of life, because it affects the mitochondrial membrane potential and protein conformational modulation (42, 43, 45).

According to the current research on laser therapy, both HILT and LLLT are commonly used, and it is possible for individuals with KOA to adopt either as a treatment in the management of pain (34, 35). However, while LLLT is adequately effective, and may be an appropriate treatment for KOA, from this review (SUCRA = 77.4%), HILT is able to penetrate tissues and joints strongly, so that it is more able than LLLT to enhance the metabolism of substances associated with pain, inflammation, and swelling (31, 34, 35), which may yield better improvement (HILT vs. LLLT on VAS pain, WMD: 0.81) (32). Admittedly, a further point is the cost of laser therapy. At present, on the market, high intensity laser is more expensive than low level laser to purchase (12, 33). Compared with low level laser, hospitals or physical therapy institutions need to spend more money to configure high intensity laser equipment, and, for patients, the cost of using HILT is also greater (33). Based on this, LLLT may be more suitable for both patients and therapists because it is more economical than HILT.

It is noteworthy that when using high intensity laser equipment for treatment, if the dose is insufficient, the resulting effect may not be as good as that with LLLT in individuals with KOA (34); so in this sense, mild dose laser therapy may not be recommended (SUCRA = 44.4%). Also, the SUCRA value of electric stimulation plus ultrasound, at 62.6%, was between that of LLLT and mild dose laser therapy, which may also have a positive effect on pain relief since the stimulation of sensory nerves by electricity can lead to an increase of the pain threshold (39).

The last three treatments were shock wave therapy, placebo laser (plus exercise), and thermotherapy plus interferential current. Shock wave therapy may be beneficial for relieving pain (38), but its effect may not be significant compared with the above treatments (SUCRA = 29.4%). Moreover, the SUCRA value of placebo laser (plus exercise) was more than that of thermotherapy plus interferential current, which indicated that it is possible that exercise therapy is more effective in relieving pain than thermotherapy involving items such as hot compress, hot pack, and heat dressing. The former improves local metabolism and blood circulation in knee joints (39), while the latter may only act on the skin, with little effect (36). Admittedly, the evidence associated with these findings was very limited (included studies were very few), thus they cannot be fully verified.

Lastly, in terms of pain management of individuals with KOA, as measured by VAS pain and WOMAC pain scores, the present meta-analysis has demonstrated that, when compared to a placebo laser (plus exercise), HILT achieved a significant improvement, with statistical significance and clear homogeneity. Notably, in the VAS pain scores of comparing HILT to control, a previous systematic review included six studies (14), and its result of meta-analysis was highly heterogeneous (*I*^2^ = 90); while this review included one more study and showed good homogeneity, indicating that the result of the current meta-analysis may have a higher credibility. Furthermore, as for pain change scores measured by whether VAS or WOMAC, not only the WMD but also the lower bound of the 95% CI was significantly more than the relevant MCID value (Table 3). These results further support the conclusion that HILT can significantly relieve pain in individuals with KOA.

### The effect of high intensity laser therapy on self-reported function

High intensity laser therapy has been widely employed in the field of physical therapy, with many studies describing its effects in alleviating pain, inflammation, and swelling (9, 14, 36), and the above has further verified the effect of HILT on pain relief in individuals with KOA. In addition, HILT was beneficial for improving physical function in KOA (31, 35, 39), and the current systematic review also shows that HILT has a significant effect on improvement in self-reported function as measured by WOMAC total score and function sub-scale. Additionally, HILT may also be superior to LLLT for improving physical function (WOMAC total, WMD: 6.48).

This outcome is probably as a direct effect of pain reduction, which makes individuals feel more comfortable and compliant in their limbs, so that physical activity becomes easier to achieve (36, 39). In addition, HILT may be beneficial for improving sport performance since short-term treatment with high-intensity laser enhances cardio-vascular function, as the activity of hemoglobin increases after being heated (30, 31, 46). Such an effect, potentially generated by HILT, is well-reflected in the function sub-scale of the WOMAC, which is largely based on questionnaire items related to physical activities such as stepping, standing, squatting, walking, and housework (28).

Judging from the SUCRA value of included physical therapy modalities, HILT may still be the best option in the management of physical function for individuals with KOA. Additionally, electric stimulation plus ultrasound may be better than LLLT in improvement of physical function, which may be due to more stimulation of neuromuscular control by electricity and thus more facilitation of limb movements (39). The next three treatments are the same as the bottom three in the ranking of the effect of HILT on pain: shock wave therapy, placebo laser (plus exercise), and thermotherapy plus interferential current.

The effect of shock wave therapy on physical function was similar to that on pain, showing a relatively low probability of improvement (SUCRA = 36.9%). Previous research has shown that shock wave has a certain effect on the enhancement of the function in KOA, but this effect may not be substantial (6, 38). Finally, the SUCRA value for placebo laser (plus exercise) was still higher than that of thermotherapy plus interferential current, suggesting that thermotherapy may not be as effective as exercise in terms of functional improvement. Hot compress applied on the skin can improve local blood circulation, but has little effect in terms of improvement in physical function (36).

Compared to placebo laser (plus exercise), when HILT was used as a physical therapy intervention for persons with KOA, improvements were evident in function, stiffness, and total of the WOMAC. However, only changes on two sub-scales and total scores were statistically significant, other differences did not exceed the relevant MCID values.

For physical function, the positive effect of HILT compared to placebo laser (plus exercise) can also be observed in the WOMAC function scale, although the lower bound of the 95% CI for the effect (6.90) was below the specified MCID value of 7.65 (29). Of particular interest here was that there was heterogeneity in the pooled studies, largely as a result of one study involving exercise that consisted of nine events (39), measured by WOMAC function, which showed significantly superior effects to other trials for individuals with KOA. Comparing with a previous study (14), the current review found the clear cause of heterogeneity within WOMAC function. This finding suggests that combining HILT with a variety of exercises may have a greater effect with respect to improving physical function in individuals with KOA (47, 48).

In terms of other symptoms of KOA, specifically joint stiffness, the present review indicated that HILT enhancements may not be at a sufficient level to be beneficial. Although this result was similar to the previous review (14), the clinical significance of HILT for stiffness has not been explained; therefore, this study further explored this phenomenon. Specifically, results here showed that gains on the stiffness sub-scale of the WOMAC were less than its relevant MCID value, despite being statistically significant. There are two possible explanations for this finding: (1) individuals treated with high intensity laser are always in a static state (37), while the improvement of stiffness typically requires range of motion training that involves repeated flexion and extension of the knee joint (44, 49, 50). Consequently, HILT does not bring about changes that promotes utilization of knee range of motion (30). (2) Most of the studies included in the review involved trials that conducted a treatment protocol with a relatively short duration, usually of 4–6 weeks, where changes in range of motion due to slower neuromuscular adaptation may not be achievable. Typically, improving knee range of motion can be achieved in 8–12 weeks (51).

Finally, HILT yielded a significant benefit in KOA, seen from using outcomes assessed by the WOMAC total, although the lower bound of the 95% CI for the effect was below the specified MCID value, which is a meta-analysis that no study has conducted so far. This gain was mainly due to the improvement of pain and function caused by HILT in individuals with KOA. Yet its effect on stiffness was not ideal, which was possibly also the reason why the values in confidence interval did not all exceed its MCID. There was heterogeneity in this result from the meta-analysis, but this arose because of the study that involved multiple exercises (39). Had this study been excluded, the results of meta-analysis would be very homogeneous (*I*^2^ = 0). This again suggests that exercise plays a vital role in the rehabilitation of individuals with KOA.

### Limitations

There are several limitations associated with this study. First, some studies may have been missed because the databases searched were limited to those listed, and there may have been some potentially relevant trials that were not included due to the search terms used. Further, the evidence needed to draw conclusions may not be sufficient as a result of the fact that only nine studies were selected for network meta-analysis, and the sample size in some trials was relatively small. In addition, the participants were not perfectly homogeneous with respect to their demographics, including age, gender, height, weight, and KOA grade; however, there is a strength in this heterogeneity, in that the results are more generalizable, and able to be applied to a wider population demographic. Also, there were differences in the high intensity laser devices and the protocols of treatment among the included studies, such as treatment position, laser wavelength, duration, and frequency. Finally, the classification of physical therapy modality may not be accurate enough. In particular, the definition of placebo laser (plus exercise) may be imprecise due to the inclusion of a variety of exercise programs, and there were some uncertain effects when a physical therapy modality included supplementary treatments such as ultrasound and interferential current.

## Conclusion

The current network meta-analysis showed that the effects of HILT may be superior to the effects obtained from other physical therapy modalities on pain and self-reported function in individuals with KOA. Given that the number of studies was limited, more high quality trials are needed to verify these findings. In addition, compared to placebo laser or exercise or a combination of both, HILT was able to relieve pain and may improve function in individuals with KOA, but may not be as clinically effective for improving knee stiffness. Clinicians working with individuals with KOA, when deciding whether to use HILT, should first determine which of pain or stiffness most need relief.

## Data availability statement

The original contributions presented in this study are included in the article/supplementary material, further inquiries can be directed to the corresponding author.

## Author contributions

MW and LL: conceptualization, methodology, formal analysis, investigation, and writing—original draft. AP, JW, RA, and JB: conceptualization, methodology, formal analysis, and writing—review and editing. JH: conceptualization, methodology, formal analysis, investigation, writing—review and editing, supervision, and funding acquisition. All authors read and approved the final version of the manuscript.

## Footnotes

1
https://www.researchregistry.com
